# Cortical Mechanisms of Central Fatigue and Sense of Effort

**DOI:** 10.1371/journal.pone.0149026

**Published:** 2016-02-09

**Authors:** Simon A. Sharples, Jason A. Gould, Michael S. Vandenberk, Jayne M. Kalmar

**Affiliations:** Department of Kinesiology, Wilfrid Laurier University, Waterloo, ON, Canada; University Medical Center Goettingen, GERMANY

## Abstract

The purpose of this study was to investigate cortical mechanisms upstream to the corticospinal motor neuron that may be associated with central fatigue and sense of effort during and after a fatigue task. We used two different isometric finger abduction protocols to examine the effects of muscle activation and fatigue the right first dorsal interosseous (FDI) of 12 participants. One protocol was intended to assess the effects of muscle activation with minimal fatigue (control) and the other was intended to elicit central fatigue (fatigue). We hypothesized that high frequency repetitive transcranial magnetic stimulation (rTMS) of the supplementary motor area (SMA) would hasten recovery from central fatigue and offset a fatigue-induced increase in sense of effort by facilitating the primary motor cortex (M1). Constant force-sensation contractions were used to assess sense of effort associated with muscle contraction. Paired-pulse TMS was used to assess intracortical inhibition (ICI) and facilitation (ICF) in the active M1 and interhemispheric inhibitory (IHI) was assessed to determine if compensation occurs via the resting M1. These measures were made during and after the muscle contraction protocols. Corticospinal excitability progressively declined with fatigue in the active hemisphere. ICF increased at task failure and ICI was also reduced at task failure with no changes in IHI found. Although fatigue is associated with progressive reductions in corticospinal excitability, compensatory changes in inhibition and facilitation may act within, but not between hemispheres of the M1. rTMS of the SMA following fatigue enhanced recovery of maximal voluntary force and higher levels of ICF were associated with lower sense of effort following stimulation. rTMS of the SMA may have reduced the amount of upstream drive required to maintain motor output, thus contributing to a lower sense of effort and increased rate of recovery of maximal force.

## Introduction

Central fatigue is a progressive reduction in the ability of the central nervous system to maximally activate muscle. Central fatigue has spinal and supraspinal origins. Supraspinal fatigue is characterized by reduced output from the motor cortex to the spinal motor neuron pool and has been suggested to contribute to reduced activation of muscle (for review see [1,2]). Others have suggested that voluntary activation is not limited by excitability of the corticospinal motor neuron and that the origin of activation failure lies upstream to the corticospinal motor neuron [3,4].

Several investigations that employ transcranial magnetic stimulation (TMS) of the primary motor cortex (M1) [1,5] suggest that corticospinal excitability is reduced for a prolonged duration when assessed at *rest* following fatiguing contractions. When assessed *during* a fatiguing maximal contraction, motor evoked potentials elicited with TMS increase and the accompanying silent period is lengthened [6], and cervicomedullary-evoked potentials (a measure of spinal motor neuron responsiveness) are depressed [7]. Studies employing ischemia (for review see [4]) and caffeine [3,8] suggest that fatigue-associated changes in cortical and spinal motor neuron excitability can be dissociated from measures of supraspinal fatigue and appear to accompany, but not cause voluntary activation failure. It is possible that hypoexcitability of spinal or cortical motor neurons could be compensated for by increased drive to the corticospinal motor neuron from upstream motor regions. There are two interesting implications of this possibility. The first is that the required increase in upstream drive may contribute to increased sense of effort. The second is that an inability to maintain adequate drive from premotor areas to the primary motor cortex may contribute to voluntary activation failure. Thus, the purpose of this study was to investigate cortical mechanisms upstream to the corticospinal motor neuron that may be associated with central fatigue and sense of effort during and after a fatigue task. Changes in the activity of inhibitory and excitatory cortical circuits within or between hemispheres may contribute to reductions in descending supraspinal drive. To address the contribution of cortical components upstream to the corticospinal motor neuron pool, the present study compared changes in intracortical inhibition and facilitation and interhemispheric inhibition following a series of contractions of the first dorsal interosseous (FDI) that elicited voluntary activation failure (central fatigue) to changes elicited by a less rigorous series of contractions that elicited peripheral but not central fatigue. The FDI was selected for study based on the role of the SMA in manual control and accessibility of the hand region of the motor cortex to TMS. We implemented subthreshold high-frequency (5Hz) repetitive transcranial magnetic stimulation (rTMS) of the SMA in an attempt to offset reduced excitability upstream to the primary motor cortex to offset voluntary activation failure during recovery from fatigue. We selected the supplementary motor area (SMA) as the target structure for upstream stimulation because it is an important source of upstream drive to M1 and sends projections to the M1 [9–12], with extensive transcallosal connectivity [13,14] and it plays a particularly important role in the control of internally-generated bimanual coordination [15,16] and unimanual muscle activation [17]. Furthermore, SMA activity is reduced following a fatiguing task [18,19] and maytherefore may be a source of reduced excitatory drive to the corticospinal motor neuron pool.

Paired-pulse TMS was used to probe the contribution of the intracortical inhibitory and facilitatory circuits that reside with layer II and III of the M1 [20]. Several studies have found that short-interval intracortical inhibition (SICI) is reduced [21,22] and intracortical facilitation (ICF) increased [21] when assessed at rest, following a fatiguing bout of contractions of muscle. In addition to circuits acting within the active M1, transcallossal circuits originating in the ipsilateral motor cortex may also play an important role in the modulation of motor output to ipsilateral muscles through both ipsilateral corticospinal projections and modulation of contralateral corticospinal output via the interhemispheric inhibitory (IHI) pathway [23]. Previous investigations have reported that reduced ipsilateral motor cortex excitability is accompanied by reduced SICI in the non-fatigued hemisphere [24], however the ability of the ipsilateral motor cortex to modulate the contralateral motor cortex via the IHI pathway following fatigue has not been explored.

Supraspinal fatigue is also associated with an increase in the perception of effort required to maintain force during a prolonged muscle contraction [25,26]. While the ability to sense limb position and muscle force involves the integration of both a corollary of the motor command and proprioceptive information provided by sensory afferents [27], it has been suggested that sense of force is strongly mediated through the integration of the central efferent motor commands by the somatosensory cortex [28]. A facilitation system proposed by Tanaka and Watanabe [29] composed of a neural circuit that connects several subcortical and cortical structures upstream from the motor cortex such as the limbic system, basal ganglia, thalamus, orbitofronal cortex and prefrontal cortex may act to offset increased inhibition and decreased excitability of the primary [2] and supplementary motor areas [18] associated with supraspinal fatigue. Increased drive from centres upstream from motor areas could in turn result in increased sense of effort due to the contribution of efferent motor command to kinesthesia (for review see [30]). If this is the case, then offsetting a fatigue-induced deficit in drive upstream to the primary motor cortex would decrease sense of effort. We therefore hypothesized that offsetting a fatigue-associated decline in premotor activity using high frequency rTMS to SMA would decrease force sensation and offset voluntary activation failure through decreased inhibition or increased facilitation of the motor cortex.

## Methods

### 2.1 Participants

Twelve healthy participants (9 males, 3 females) took part in this study (mean±sd; 21.8±2.5 years). All participants were non-smokers and right-handed (confirmed using the Waterloo Handedness Questionnaire) [31]. Participants were excluded if they had previous diagnosis of a neurological disorder, neural symptoms in the upper extremities (unexplained numbness, coldness, or tingling), or contraindications to TMS [32] self-reported via questionnaire. Participants were asked to avoid consumption of caffeine products twelve hours prior to each experimental session, and were told to reschedule their session if they were ill or slept poorly the night before the experiment. The study was approved by the Wilfrid Laurier University Research Ethics Board (approval # REB2440) and is in accordance with the Declaration of Helsinki. All participants provided written informed consent. All TMS protocols comply with previously published safety recommendations [32].

### 2.2 Experimental design

In this repeated measures study, participants attended 4 sessions on 4 separate days. On two of the days, participants performed a rigorous bout of maximal and submaximal isometric contractions of the right FDI meant to elicit central fatigue. On one of these central-fatigue days, 5Hz rTMS was applied to the SMA at task failure (defined below) in attempt to offset fatigue- associated reductions in SMA excitability [33] and hasten recovery. The other two days were control days when participants performed intermittent maximal isometric contractions of the right FDI that did not elicit central fatigue. On one of these control days, 5Hz rTMS was delivered to the SMA at the end of the task. On the no-stim days, participants sat quietly for the 5-minute time period with the stimulation protocol delivered but the coil hovering 3-5cm above the participant’s scalp. The order of these 4 experimental days (fatigue+stim, fatigue+no stim, control+stim, and control+no stim) was randomized and counter-balanced. To accomplish this, 12 strips of paper, each with a prescribed order of the 4 experimental sessions were prepared in advance of the investigation. Each of the 12 participants drew one strip of paper from an envelope at the beginning of their first experimental day.

### 2.3 Experimental protocol *(Fig 1)*

#### Baseline Measures

**Fig 1:**
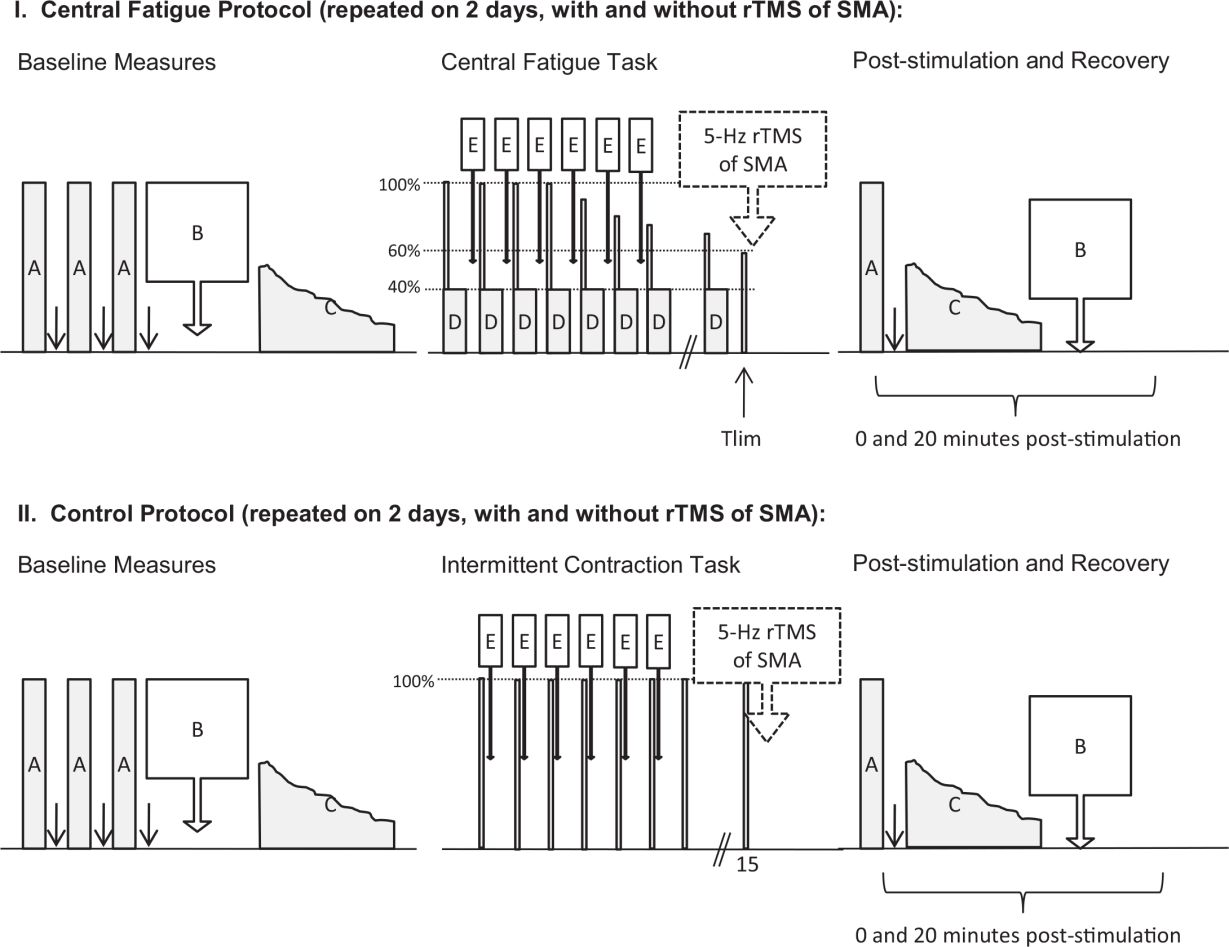
Experimental Paradigm. The experimental protocol was repeated on 4 separate days. Two days included a central fatigue task that involved a series of 10 isometric contractions of the right FDI at 40% MVC (each set of 10 contractions is denoted by one box labeled D). On two days, the fatigue task was replaced with a rest period to provide a “no-fatigue” control. On one fatigue day, and on one control day, 5-Hz stimulation was omitted to provide “no-stimulation” controls. These days are not shown in the figure, but are identical aside from omission of the dashed boxed arrow denoting 5-Hz rTMS. During the baseline and recovery periods, the following measures were made: voluntary activation assessed during maximal contractions of the right FDI (A), cortical excitability and inhibition assessed with TMS (B) and sense of effort (C). During the baseline period the order of these measures were set to prevent fatigue. During the recovery period, the order of these measures was set to minimize recovery in the interval between assessing voluntary activation and cortical excitability. During the fatigue task (and control day rest periods), single measures of cortical excitability, inhibition and maximal voluntary activation were assessed every minute (E).

**Fig 2:**
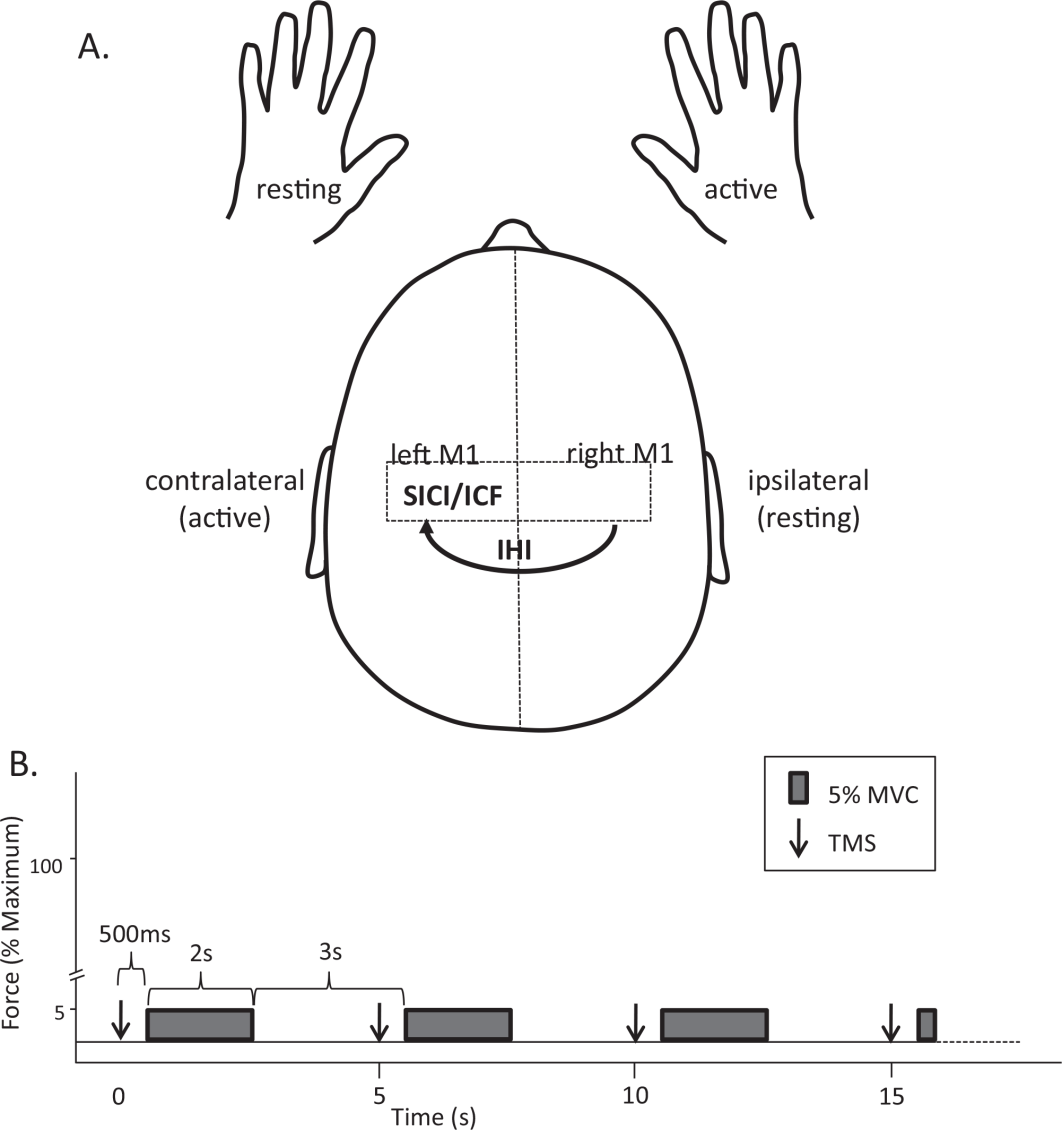
Paradigm for Transcranial Magnetic Stimulation. Cortical excitability, intracortical inhibition (SICI) and intracortical facilitation (ICF) were assessed within the left (active) motor cortex and interhemispheric inhibition (IHI) was assessed from the right (resting) to the left (active) 500ms prior to a low level contraction of the right FDI (A.). Measures of cortical excitability and inhibition were made 500ms prior to rhythmic low-level isometric contractions (5% MVC) of the right FDI. Contractions were maintained for 2 seconds with 3 seconds rest between contractions to prevent neuromuscular fatigue. The frame-based software allowed static cursors to be placed at 500ms and 2.5s to be used as cues to contract and relax the right FDI. This allowed for contractions to be rhythmic and predictable in nature Magnetic stimuli are represented as downward arrows and were applied at the start of each frame. (B).

**Fig 3:**
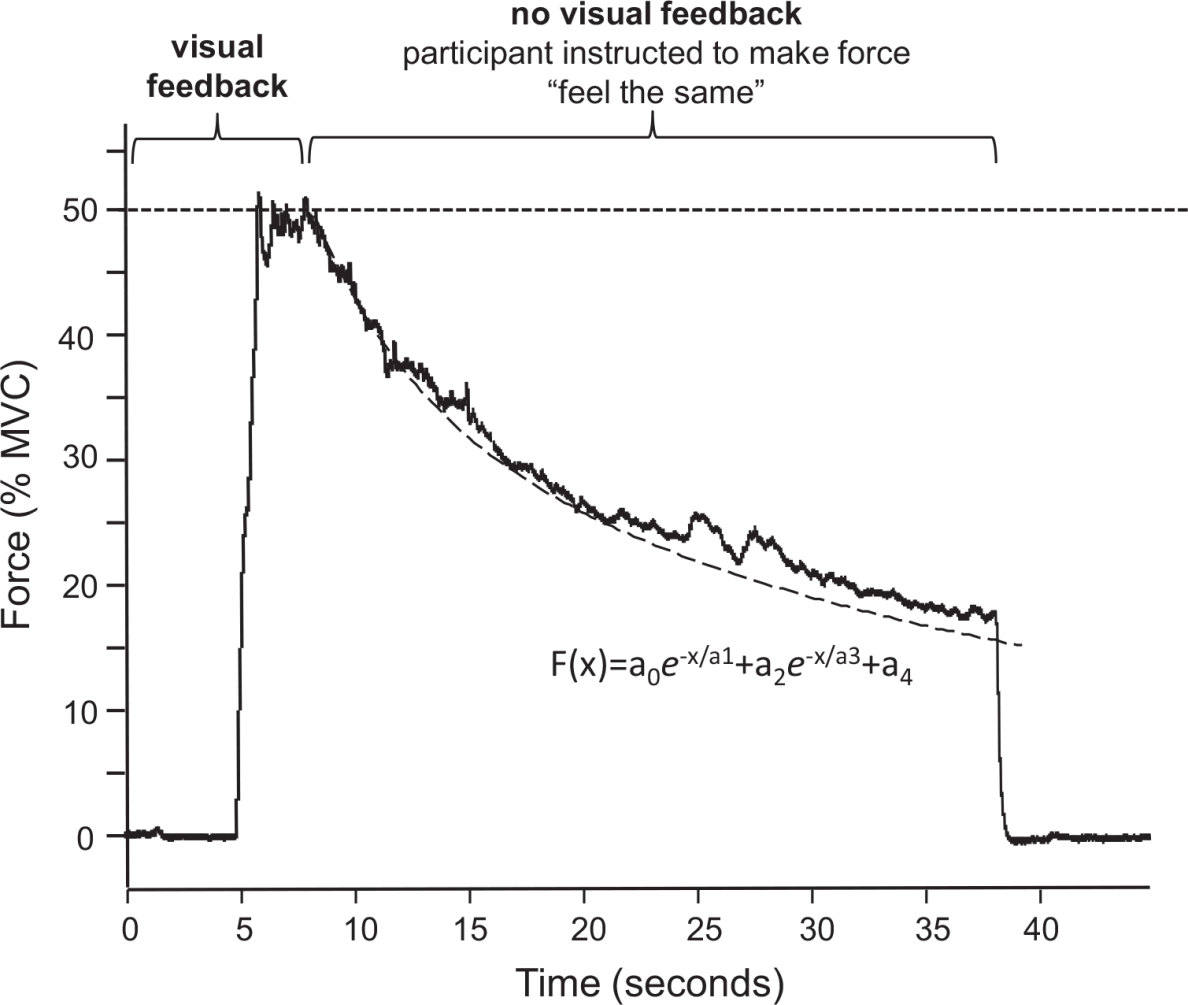
Constant Force Sensation Contractions. Sense of force was measured using a constant sense of effort contraction of the right FDI. Participants had 5 seconds to view the target force and prepare to contract and 3 seconds to reach and hold force at the 50%-MVC target. Visual feedback of force and the target were then removed and participants were instructed to continue the contraction for 30 seconds but to adjust their force as required to “make the force feel the same”. The resultant decline in force is fitted to a double exponential function (black dashed line and inset equation) yielding two rate constants (a1 and a3) that are used as a measure of sense of effort. Percent decline from the start to finish of the contraction is also used to index the amount of effort required to sustain the contraction.

At the start of each session (Fig 1), included 3–5 maximal voluntary contractions (MVC). An electrical pulse was applied to the ulnar nerve during and 2 seconds after each MVC to quantify the voluntary activation according to the twitch interpolation technique. Participants rested for at least 1 minute after each attempt to reduce fatigue at baseline. A paired-pulse TMS protocol was then performed to assess intracortical facilitation and inhibition, and interhemispheric inhibition 500ms prior to a low-level unimanual contraction (Fig 2) [34]. Following the TMS protocol, participants performed a constant force-sensation contraction (Fig 3; described in section 2.6) [35].

#### Fatigue Task

A finger abduction fatigue task was developed based on one previously shown to elicit central fatigue [5] and further developed through pilot work to optimize central fatigue using contractions that are submaximal and intermittent while minimizing peripheral fatigue. This fatigue task began immediately following baseline measures of force sensation. For the central fatigue task (top panel, Fig 1), each set of contractions in the fatigue protocol included one MVC of the right FDI (during which time voluntary activation was assessed) followed by 10 submaximal contractions at a target force of 40%-MVC. Each contraction was held for 3s with a 2s-rest between contractions. The target force was displayed on a computer monitor for the participant and verbal encouragement to stay on the target was provided by the investigator throughout the experiment. Following each set of contractions, single measures of SICI, ICF, and IHI were made using paired-pulse TMS. Each set of contractions (1 maximal and 10 submaximal contractions) took approximately 1 minute to complete. Sets were repeated to the point of task failure (T_lim_), which was defined as a 40% reduction in MVC force. On the control day (bottom panel, Fig 1), participants performed 1 MVC every minute for 15 minutes. Single measures of intracortical inhibition, intracortical facilitation and interhemispheric inhibition were made using paired-pulse TMS after each MVC. At the end of the fatigue task, 5-Hz stimulation was applied to the SMA (as described below).

#### Post-stimulation and Recovery Measures

Immediately (0 minutes) and 20 minutes after 5-Hz stimulation of the SMA (or after the equivalent time period on the no-stimulation days), one maximal voluntary contraction, force-sensation, and paired-pulse stimulation were reassessed.

### 2.3 Experimental apparatus and data acquisition

Participants were seated in a modified automobile seat with the left and right forearm secured in thermomoldable splints mounted on armrests. Elbows were positioned at approximately 90°, forearms were pronated, and the thumb and index finger were maintained at a constant angle of 60°. Surface electromyography (EMG) was recorded from the left and right FDI using silver-silver chloride electrodes that were epoxy-embedded with a preamplifier (10x) (EQ Inc., Chalfont, PA). Recording surfaces were 0.8 cm in diameter and were spaced 2 cm apart over each muscle belly. Preamplifiers fed into a variable-gain second stage amplifier (20x) for a total gain of 200x. Force was a recorded with a force transducer strapped to the lateral aspect of the proximal interphalangeal joint of the second digit with the hand secured comfortably in a custom-built dynamometer. Force was amplified with a custom-built amplifier (10x). Experimental protocols were sequenced and data was collected using frame-based data acquisition software (Signal, Version 6, Cambridge Electronics Design, UK). EMG and force signals were digitized at 2 kHz (Micro3 1401, Cambridge Electronics Design, UK). Surface EMG was band-pass filtered from 10 to 1000 Hz and force was low-pass filtered at 50 Hz. Signals were stored on a laboratory computer for offline analysis.

### 2.4 Electrical stimulation of the ulnar nerve

The ulnar nerve was stimulated using a 1cm^2^ cathode positioned immediately lateral to the pisiform bone of the wrist and a 2.5cm^2^ anode secured to the dorsal aspect of the forearm, just proximal to the wrist. Both electrodes were made of carbonized rubber, coated with electroconductive gel and held in place using adhesive medical tape and a tensor bandage. Square pulses with a 200μs pulse duration were applied with an electrical stimulator (Digitimer, model DS7A, Hertfordshire, UK) at a current intensity of 110% of that which was sufficient to evoke a maximal twitch.

### 2.5 Transcranial magnetic stimulation (TMS)

TMS of the left motor cortex was performed using a 70mm double cone coil connected to two Magstim 200 magnetic stimulators via a Bistim module (Magstim Company, UK) with the handle positioned posteriorly 45°to the midsagittal line and the induced current in a posterior to anterior direction. Optimal coil position was determined by moving the coil in 1cm increments around the presumed FDI hotspot in the M1. The location that elicited finger abduction and the largest motor evoked potential (MEP) was considered optimal and was digitized and saved using a coil tracking system (TMS Navigator, Northern Digital Instruments, Waterloo, CAN). This experiment required the use of two coils, one to stimulate each hemisphere to assess interhemispheric inhibition. Coil position for the right motor cortex was determined using the same method as the left motor cortex. Single magnetic stimuli were delivered through a 70mm double cone coil over the right hemisphere using a Magstim Rapid^2^ Stimulator (Magstim Company, UK).

#### TMS motor thresholds

Stimulation intensities were set relative to motor thresholdsthat were determined at the beginning of each experiment. Resting motor threshold (RMT) was determined by delivering single monophasic magnetic stimuli to the left motor cortex and was defined as the minimum stimulator output required to elicit an MEP ≥ 50μV peak-to peak in amplitude in 5 out of 10 trials. Motor threshold was also measured at rest 500ms prior to the onset of an anticipated isometric contraction, an approach we have previously used to study modulation of motor circuits when muscle is at rest, but premotor structures may be active [34]. This threshold was determined using the same criteria as at rest and is referred to as “pre-contraction motor threshold.” Resting motor threshold was also measured in the left and right motor cortex using single biphasic magnetic stimuli applied through the Rapid^2^ Air-Cooled coil at the start of each session and used to determine rTMS stimulation intensity. Motor thresholds assessed using the Rapid^2^ and Bistim are presented in Table 1 as mean±sd.

**Table 1:** Motor threshold was assessed at the beginning of each experiment on each day with the muscle at rest (RMT) and at rest but 500ms prior to a contraction (Pre-contraction motor threshold (PCMT)) using a single monophasic magnetic pulse from the Bistim2 stimulator over the left motor cortex. Resting motor threshold was also assessed in the left and right primary motor cortex using the Rapid^2^ stimulator and are presented as average between the two hemispheres*. Motor thresholds were consistent at baseline on all 4 days. Bolded averages across the four days indicate that motor threshold was lower when assessed prior to a contraction (PCMT) compared to at rest† and higher using a biphasic magnetic stimulus from the Rapid^2^ stimulator††. Data are presented as mean±sd.

Day	RMT (Bistim)	PCMT (Bistim)	RMT (Rapid^2^)*
Fatigue+Stim	41.9±5.6	39.5±6.1	47.9±5.1
Fatigue+No Stim	41.9±4.4	40.2±5.7	47.8±4.7
No Fatigue+Stim	42.2±4.8	40.4±4.9	47.1±5.0
No Fatigue+ No Stim	41.5±4.9	39.5±4.9	46.6±4.9
**Average**	**41.9±4.5**	**39.9±5.2†**	**47.3±4.8††**

#### Paired-pulse TMS protocol

Paired-pulse TMS was used to assess interhemispheric inhibition of the fatiguing motor cortex by the resting motor cortex and intracortical inhibitory and facilitatory circuits within the fatiguing motor cortex (Fig 2A). We assessed SICI, ICF, and IHI with the muscle at rest, 500ms prior to an anticipated contraction of the right FDI. This “pre-contraction” TMS stimulation protocol was used because it provides a measure of cortical excitability during a period when premotor structures may contribute to the unimanual task but while the muscle is still at rest to avoid confounding effects of fatigue-induced changes in muscle activation on the motor evoked potential over the course of the protocol [34]. For the paired-pulse protocol, participants performed a series of brief (2.5-s), low-level contractions (5%-MVC) cued by a vertical cursor displayed on a computer monitor for force feedback (Fig 2B). Paired pulses to assess SICI, ICF, and IHI were applied in a 500-ms period before each contraction. SICI was assessed by delivering a subthreshold conditioning stimulus 2ms prior to a suprathreshold test stimulus through the same coil [20]. ICF was assessed by delivering a subthreshold conditioning stimulus 10ms prior to a suprathreshold test stimulus through the same coil [20]. The conditioning stimuli to elicit both SICI and ICF were set to 80% of the pre-contraction motor threshold. IHI was assessed by delivering a suprathreshold conditioning stimulus to the ipsilateral (right) hemisphere (contralateral conditioning stimulus, CCS10), 10ms prior to the test pulse to the left hemisphere. The conditioning pulse delivered to the contralateral hemisphere to elicit interhemispheric inhibition was adjusted to a stimulator output to evoke a MEP of 1mV peak-to-peak amplitude in the left FDI [36]. All stimulation parameters utilized to assess interhemispheric inhibition, intracortical inhibition and intracortical facilitation were adjusted to elicit 1mV MEPs in the left and right FDI when elicited within a 500-ms period prior to a contraction. Ten trials of the test MEP, SICI, ICF, and IHI were randomized for a total of 40 trials at each point of assessment (baseline, post and recovery). Single measures of each were also made following each set of unimanual contractions in the fatigue protocol or every minute in the non-fatigue protocol.

#### Repetitive Transcranial Magnetic Stimulation of the SMA

We employed high frequency (5Hz) rTMS to increase the excitability of the SMA. Repetitive TMS was performed using a 70mm double air-film coil coil connected to a Rapid^2^ Magnetic stimulator (Magstim Company, UK). The SMA was stimulated by positioning the coil 3cm anterior to vertex with the handle pointing posteriorly and in line with the midsagittal line to activate the supplementary motor area bilaterally [33,37,38]. A total of 1200 pulses were delivered to the supplementary motor area during a single rTMS session. The pulses were delivered using 24 trains of 50 pulses with an inter-train interval of 5 seconds. Stimulation intensity was set to 90% of the average RMT of both the left and right of FDI (thresholds determined using the Rapid^2^ Air-Cooled coil attached to the Rapid^2^ stimulator) [33]. We also employed a non-stimulation control day with the stimulation protocol delivered in the same position as the stimulation day but with the coil hovering 3-5cm above the participant’s scalp control for noise, distraction, and time-dependent changes

### 2.6 Constant force-sensation contractions

Participants were instructed to contract the right FDI to a target force of 50% MVC while visual feedback was provided on a computer monitor. Participants were given 3s to reach and maintain the target force during which time they were to concentrate on their perceived effort required to maintain this force by the FDI muscle. Visual feedback was then removed. The participants were instructed to hold the contraction for another 30 seconds at what *felt like* a constant force. The result is a very reproducible decline in force (Fig 3) which provides a continuous measure of the effort associated with a sustained muscle contraction, such that a greater rate of decline in force indicates a greater sense of the effort associated with producing force [35,39].

### 2.7 Data Analysis

Average MEPs from ten trials were generated for each condition (ie, TS, SICI, ICF, IHI) measured in the right FDI at three time points (baseline, 0-min post-stimulation, and 20min recovery) using frame-based software (Signal, Cambridge Electronics Design). Prior to averaging, each frame was visually inspected to ensure that there was no muscle activation during the 500ms window prior to contraction onset when the stimuli were delivered. For all TMS measures, frames were excluded if activation was detected in the left FDI. Peak-to-peak MEP amplitudes are reported in millivolts (mV). Conditioned MEP amplitudes are reported as a ratio between the conditioned MEP and the unconditioned test pulse MEP amplitude. Stimulator output intensities are reported as percent of the maximal stimulator output (%MSO). Root mean square was used as an amplitude measurement of electromyographical activity during voluntary contractions. The amplitude of the EMG during the maximal contractions is expressed as a percent of baseline maximal RMS of the EMG. Maximal voluntary activation was assessed using the twitch interpolation technique [40]. Voluntary activation was calculated with a standard twitch interpolation equation:

$$\%\;Voluntary\;Activation=\left(1-\frac{SIT}{POT}\right)\;*\;100$$

where SIT is the superimposed twitch generated by applying the supramaximal electrical pulse during a voluntary contraction, and POT is the potentiated twitch elicited at rest, approximately 2 seconds after the contraction. The force recording during the constant sense of effort contraction was fitted in Signal (Signal, Version 6, Cambridge Electronics Design, UK) to a double-exponential defined by the function:

$$F\left(x\right)=\left(a0\;e^{-\frac{x}{a1}}\right)+(a2\;e^{-\frac{x}{a3}})+\;a4$$

This function provides two time constants (a1 and a3) that describe the two distinct rates of decline in force and serve as a measure of sense of effort. A larger time constant is the result of force declining over a longer time and is indicative of a lower sense of effort. To make this measure more intuitive, we present the inverse of the time constants so that a larger constant represents a higher sense of effort. The percent decline (% decline) in force from the beginning to the end of the contraction was also measured as a measure of sense of effort (for example, see Fig 3). All data are reported as mean ± sd.

### 2.8 Statistical analysis

A power calculation was conducted comparing differences in the normalized test MEP amplitude at Tlim between both control and fatigue days for each participants. This analysis revealed a power of the test to be 0.997. A post hoc sample size calculation on voluntary activation data revealed significant reductions (p<0.05) with a power of 0.9 to be detected with as few as 8 participants.

Single measures of voluntary activation, test MEP, intracortical inhibition, intracortical facilitation and interhemispheric inhibition that were made after each set during the progression of the fatigue protocol were pooled across the days that participants received stimulation to the supplementary motor area and averaged over the two fatigue and two non-fatigue days. Because the number of sets completed by participants varied greatly, fatigue-associated change in MVC, maximal RMS, M-wave and contractile properties (PT, TTPT, ½ RT) and measures of cortical excitability were assessed by comparing the baseline measure to the measures made at Tlim. This was conducted using a2x2 repeated measures ANOVA with fatigue day (fatigue and no fatigue) and time (baseline and T_lim_) as within-participants factors.

The effect of rTMS to the supplementary motor area following fatigue was determined with a 2x4 repeated measures ANOVA with day (stimulation to the supplementary motor area and no stimulation) and time (baseline, T_lim_, post and recovery) as within participant factors. Pearson correlations were performed following the stimulation period on the fatigue days to examine associations between cortical excitability and inhibition with voluntary activation failure and increased sense of effort with fatigue. Post hoc analyses were performed when there was a main effect or interaction using a Fischer’s LSD. Significance was set at an alpha value of 0.05.

## Results

### 3.1 How consistent were measures of excitability at baseline?

Corticospinal excitability was assessed in the left motor cortex at baseline on all four days of testing. Three of these measures were based on stimulator output at motor threshold. These included 1) the Bistim2 stimulator output at motor threshold in the left motor cortex when the hand was at rest, 2) Bistim2 stimulator output at motor threshold in the left motor cortex 500ms prior to contraction of the right FDI, and 3) Rapid2 stimulator output at motor threshold averaged for the left and right motor cortex when the hands were at rest (Table 1). There were no differences in these baseline measures of cortical excitability across days. A fourth measure of corticospinal excitability at baseline was the stimulator output required to elicit MEPs that were 1mV in peak-to-peak amplitude (Table 2). Unconditioned MEPs were elicited in the right FDI (TS1mV) by applying single monophasic pulse from the Bistim2 stimulator to the left motor cortex. The intensity of interhemispheric conditioning stimuli was set to elicit a 1-mV MEP in the left FDI when a single biphasic pulse was applied to the right motor cortex via the Rapid2 stimulator. Stimulator output required to elicit this 1-mV MEP on the Fatigue+No stim day was slightly elevated (F(3,33) = 3.04, p<0.05). All other outputs and MEP amplitudes were consistent across days.

**Table 2:** Stimulator output was adjusted at the start of each experiment to elicit unconditioned MEPs of 1mV in the left and right FDI with the muscle at rest but 500ms prior to a low-level unimanual contraction of the right FDI. Unconditioned test MEPs of 1mV were elicited in the right FDI by applying single monophasic magnetic stimuli to the left motor cortex (M1) using a Bistim module (Test Pulse). Conditioning MEPs of 1mV were elicited in the left FDI by applying single biphasic magnetic stimuli to the right M1 to activate the interhemispheric inhibitory pathway using a Rapid^2^ stimulator (Conditioning Pulse). Stimulator intensities and elicited MEPs were matched across days however the stimulator output required to elicit 1mV conditioning stimuli on the Fatigue+No stim day was slightly elevated indicating reduced excitability of the ipsilateral M1 at baseline. Data are presented as mean±sd.

	Test Pulse (Left M1)		Conditioning Pulse (Right M1)	
Day	Stimulator Output (%MSO, Bistim)	MEP Amplitude (mV)	Stimulator Output (%MSO, Rapid^2^)	MEP Amplitude (mV)
Fatigue+Stim	45.7±6.6	1.2±0.6	63.7±6.2	0.97±0.4
Fatigue+No Stim	47.7±6.3	1.17±0.5	67.4±6.9*	1.1±0.8
No Fatigue+Stim	45.8±5.5	0.97±0.35	65.2±8.3	1.05±0.4
No Fatigue+ No Stim	45.1±7.0	1.28±0.45	64.3±7.5	1.17±0.75

* indicates a significance (Fishers LSD post hoc, p<0.05).

### 3.2 Did the fatigue protocol elicit central fatigue?

Participants performed the same number of sets until task failure on both fatigue days (Fatigue+Stim: 24.3±9.6 Fatigue+NoStim: 21.6±12.6; paired t-test p = 0.17). Because the fatigue protocol was conducted before rTMS was applied to the supplementary motor area, data were pooled across the two stimulation days to determine the effect of fatigue on MEP, SICI, ICF and IHI. Maximal voluntary force (F(1,23) = 136.6, p<0.001) declined to a greater extent on the fatigue days than on the control days and recovered with rest (Table 3). The decline in peak twitch tension (F(1,23) = 4.2, p = 0.052) (Table 3) indicates that peripheral failure may have contributed to the reduction in MVC on both days. However, only the fatigue protocol was associated with a significant reduction in maximal voluntary activation (F(1,23) = 7.2,p = 0.01) indicating that the more rigorous series of maximal and submaximal contractions elicited central fatigue (Table 3).

**Table 3:** Changes in maximal force, EMG, voluntary activation, M wave, muscle twitch characteristics, and cortically evoked potentials from baseline to post-fatigue (at Tlim) prior to the application of rTMS to the supplementary motor area or no-stimulation control period. F and p values from 2 factor repeated measures ANOVA are displayed for interaction between day (fatigue, control) and time (baseline, Tlim). In this analysis, the days with and without rTMS stimulation were collapsed (as all measures shown in this table were assessed prior to rTMS stimulation).

	Fatigue		Control		Interaction	
	Baseline	Tlim	Baseline	Tlim	F (1,23)	p
**Maximal contractions:**						
Max. Torque (N)	42.9±7.2	25.3±5.0*	42.0±9.7	36.4±8.6*	137.6	<0.001
Max.l EMG (mV)	1.3±0.39	0.83±0.26*	1.35±0.46	1.1±0.4*	13.2	0.001
% Max. Vol. Activation	96±3.9	79±16.4*	96±3.8	91±11.3	7.2	0.01
**Peripheral Nerve Stimulation:**						
M-wave (mV)	20.6±6.4	19.5±7.4	20.5±6.3	20.8±7.4	3.2	0.049
Twitch peak tension (N)	2.8±0.9	1.9±1.3*	2.7±1.2	2.3±1.1*	4.2	0.052
Time to peak tension (ms)	105±14.0	96±28.0	97±8.6	98±11.0	2.1	0.15
Half relaxation time (ms)	80±7.7	80±7.0	79±8.0	79±7.2	0.01	0.91
**Cortical Stimulation:**						
MEP/M-wave	0.06±0.02	0.02±0.01*	0.06±0.02	0.04±0.03*	5.4	0.03
Ipsilateral MEP (mV)	0.91±0.4	0.85±0.6	1.1±0.6	1.2±1.0	0.38	0.5
SICI	0.49±0.2	0.89±0.76*	0.5±0.3	1.0±0.9*	0.19	0.67
ICF	1.3±0.65	2.2±2.1*	1.3±0.4	1.9±2.3	0.35	0.56
IHI	0.47±0.3	0.7±0.5	0.55±0.4	0.93±1.17*	0.34	0.57

* indicates a significant difference from baseline within each day (Fishers LSD post hoc, p<0.05).

### 3.3 What were the cortical mechanisms of central fatigue?

Cortical stimulation revealed a significant decline in the amplitude of MEPs evoked by stimulation of the left motor cortex at task failure on the fatigue days as well as the control days (Table 3). Because the fatigue has been reported to elicit a decline in the M wave, the MEP was normalized to the M wave to account for change in peripheral transmission [5]. Intracortical inhibition was reduced at task failure on both the fatigue days as well as the control days. Intracortical facilitation was increased on the fatigue days only and interhemispheric inhibition was reduced on the control day (Table 3).

**Fig 4:**
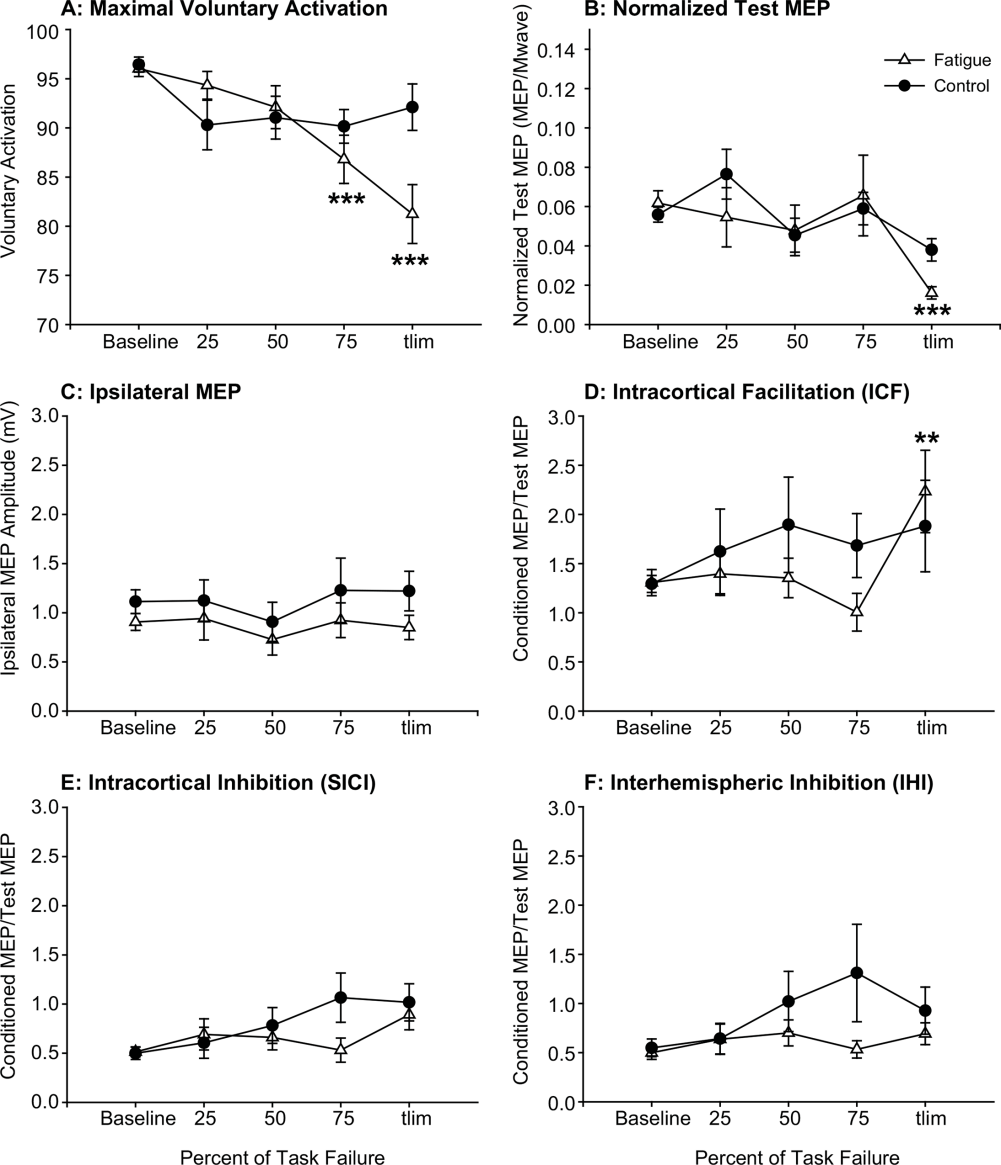
Changes in Cortical Circuitry During Fatigue. Voluntary activation (A) and normalized test MEP (B) declined over the course of the fatigue protocol and were significant at task failure. The ipsilateral MEP (C) did not change and intracortical facilitation (D) was signicantly reduced at task failure on the fatigue days. Intracortical (E) and interhemispheric inhibition (F) did not significantly change over the course of the contraction protocols on the fatigue and no fatigue control days. The days with and without rTMS were pooled in this analysis because rTMS was applied after these measures were made (at task failure). Data are represented at mean±S.E asterisks denote a significant difference from baseline measures when with a Fisher LSD when RM ANOVA was significant. p<0.05, **, p<0.01, ***p<0.001.

Single measures of voluntary activation, corticospinal excitability, intracortical inhibition and facilitation, and interhemispheric inhibition were assessed between every set of contractions on the fatigue days and at time points corresponding to the first 15 sets on the control days. Voluntary activation (Fig 4A), the MEP of the fatigued motor cortex (Fig 4B), the MEP of the non-fatigued ipsilateral motor cortex (Fig 4C), and intracortical facilitation (Fig 4D), intracortical inhibition (Fig 4E) and interhemispheric inhibition (Fig 4F) of the left motor cortex are shown over the course of the fatigue protocol. When time to fatigue is normalized (25%, 50%, 75%, and 100% Tlim) no systematic changes are evident in these data.

### 3.4 Is the recovery from central fatigue hastened by high frequency stimulation of the supplementary motor area?

**Fig 5:**
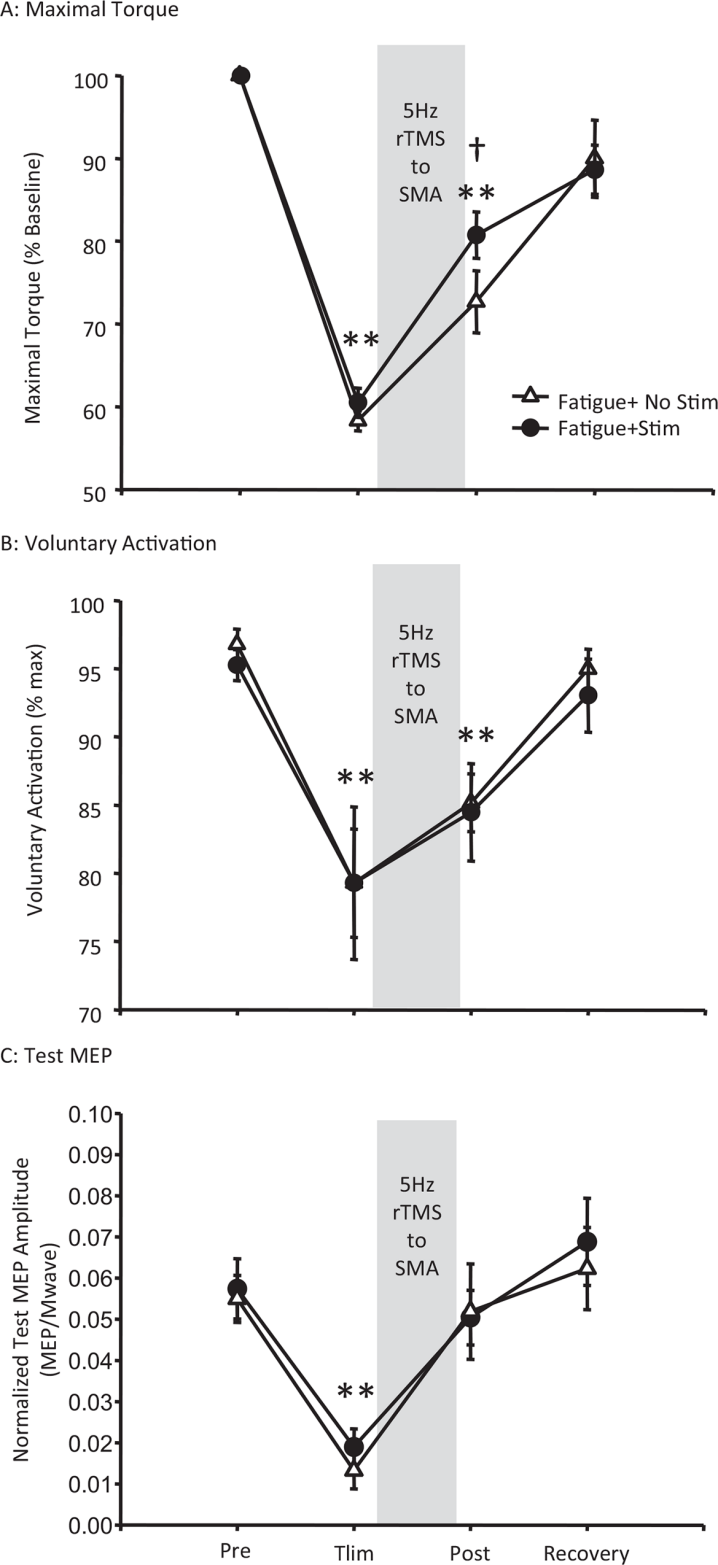
Effect of 5Hz rTMS over the SMA on Recovery from Fatigue. Maximal torque (A), maximal voluntary activation (B) and test MEP amplitude (C) were significantly reduced at task failure however rTMS to the supplementary motor area (SMA) increased the rate of recovery of maximal torque but not maximal voluntary activation or MEP amplitude. Data are presented as mean±S.E on a fatigue day where stimulation was applied to the supplementary motor area (circles) or no stimulation control day (triangles). ** indicate significant differences from baseline on both days. † indicate significant difference between days (Fishers LSD post hoc, p<0.05).

**Fig 6:**
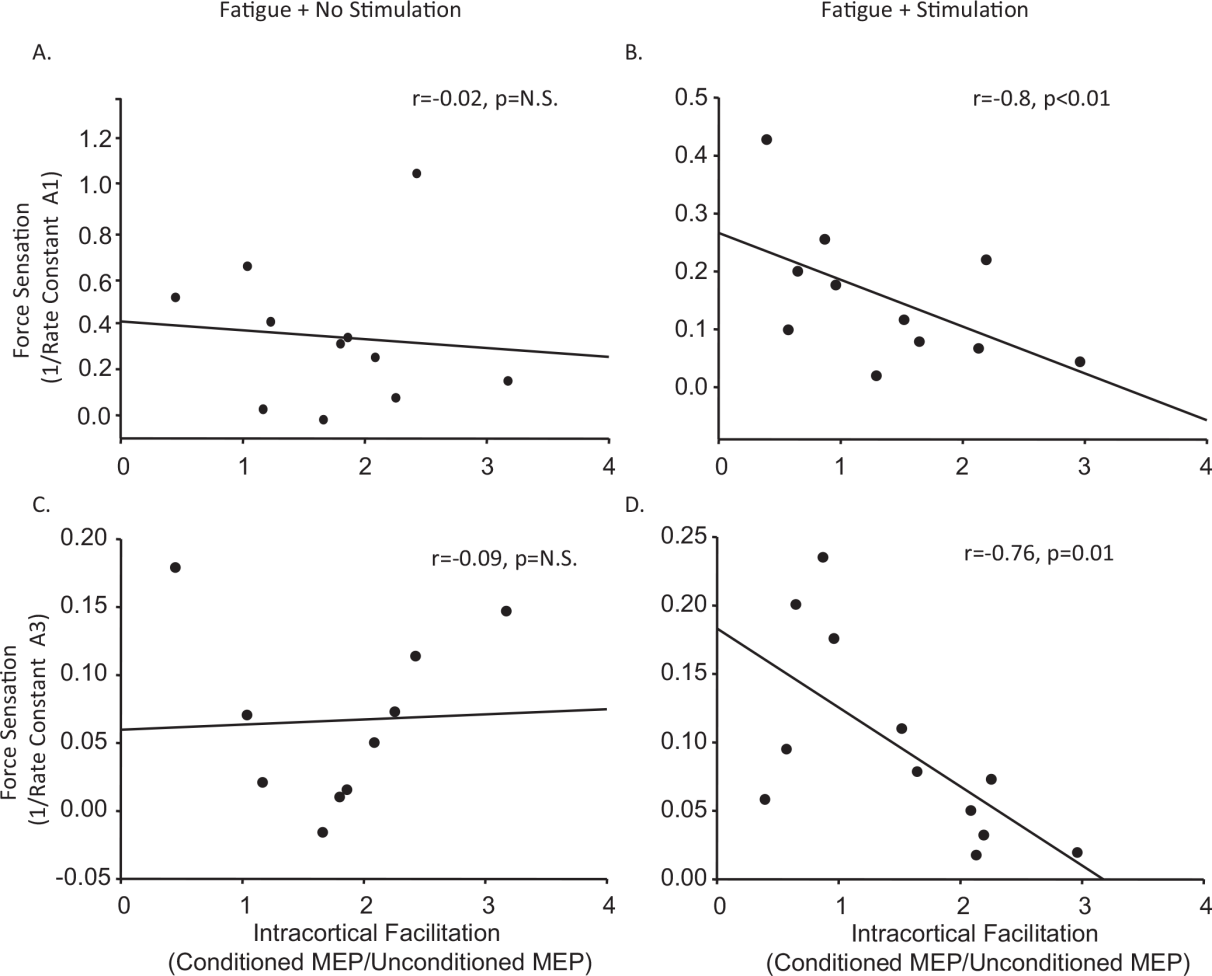
Effect of 5Hz rTMS over the SMA on the Relationship between ICF and Sense of Effort Following Fatigue. Following fatigue, and in the absence of rTMS, there was no relationship between intracortical facilitation and sense of force using the first (a1;C) or second(a3; A) rate constants of the sense of effort contraction however a strong relationship developed following high frequency rTMS of the supplementary motor area (B&D). Sense of force is displayed on the y axes using either the inverse of the first (a1) or second (a3) rate constants of the sense of effort contraction where a lower value represents increased rate of decline and greater sense of force. Intracortical facilitation is displayed on the x axis as the ratio between conditioned MEP and unconditioned MEP.

We assessed the effect of rTMS to the supplementary motor area on fatigue-induced changes in MVC, voluntary activation, sense of effort and cortical excitability and inhibition. MVC and voluntary activation were both significantly reduced at task failure and returned to baseline measures with rest, however MVC recovered faster on the day stimulation was applied to the supplementary motor area (F(3,33) = 77.7, p<0.001) (Fig 5A). This difference was not reflected in changes in voluntary activation (Fig 5B). High frequency rTMS of the supplementary motor area had no effect on the recovery of measures of corticospinal excitability (Fig 5C), intracortical inhibition or facilitation. While rTMS had no effect on overall measures of sense of effort (Data not shown), following stimulation, a relationship emerged whereby greater intracortical facilitation was associated with a lower sense of effort measured using both the first rate constant (a1) (r = -0.76, p = 0.01) and second rate constant (a3) (r = -0.8, p<0.01) of the constant-sensation contractions. This is a relationship that was not apparent at baseline or following fatigue in the absence of stimulation of the supplementary motor area (Fig 6B & 6D).

## Discussion

Previous reports have suggested that declines in voluntary activation of muscle during fatigue are not limited by corticospinal excitability and that the origin of activation failure may be upstream to the corticospinal motorneuron pool [3,8,41]. In this study we investigated the contribution of changes in cortical circuitry upstream from the corticospinal motor neuron that are associated with fatigue-induced declines in the ability to activate muscle and generate force.

### 4.1. Changes in cortical circuitry associated with central fatigue

We compared the changes in motor cortex excitability that occur with central fatigue to changes in excitability that occur during a series of less-fatiguing intermittent voluntary contractions that did not elicit central fatigue. Similar to previous investigations, the unconditioned test MEP [2,42,43] and intracortical inhibition were reduced [21,22] and intracortical facilitation was increased [21] when assessed in the cortical representation of the fatigued muscle at task failure. However, Maruyama et al [21] found that the increase in intracortical facilitation was eliminated when stimulator output was increased to maintain a constant test MEP amplitude during their fatigue protocol. On this basis, enhanced intracortical facilitation has been deemed an artifact of reduced test MEP amplitude during fatigue [21]. It is important to note that reduced MEP amplitude with fatigue may be due to reductions not only in cortical excitability, but also spinal excitability [44] or peripheral transmission failure [5]. As such, a reduction in MEP amplitude due to hypoexcitability downstream to the cortical stimulus would not warrant the adjustment of stimulator output. The reduction in intracortical inhibition observed at task failure in this and other [21,22,45] studies, and the increased facilitation that we observed in the present study may act as mechanisms of compensation and serve to offset a reduction in corticospinal excitability and downstream failure.

The necessity of selecting arbitrary time points to assess mechanisms of neuromuscular fatigue contributes to the disparity between results in the fatigue literature. Fatigue may be elicited with a sustained contraction or a series of contractions over a fixed period of time [21,24,44], resulting in between-subject variability in extent of fatigue elicited by the protocol. Conversely, measures may be made prior to fatigue and after an arbitrary point of task “failure”, with between-participant variability in the time it takes to reach this point. Furthermore, fatigue is a progressive event and cortical and spinal adaptations are not necessarily linear as they are the product of activation history that may potentiate the system, as well as failure and compensation as the task continues. This is particularly true of fatigue-associated changes in cortical excitability which demonstrate a potentiation at the onset of a fatigue protocol, followed by a decline following the cessation of the fatigue protocol [5,43]. In the present investigation, we assessed single measures of cortical excitability and inhibition during brief pauses between sets throughout a fatigue protocol that was terminated when maximal force declined by 40% and that elicited central fatigue (reduced maximal voluntary activation). Voluntary activation and unconditioned test MEP amplitude assessed between sets appeared to decline with increasing number of sets completed. Although intracortical inhibition decreased and intracortical facilitation increased from baseline to Tlim, this was not evident when assessed between sets over the course of the fatigue protocol. Based on the changes that occurred from baseline to Tlim (Table 3), we expected to see progressive changes in cortical excitability between sets. However, when time to fatigue was normalized (e.g. 25%, 50%, 75%, and 100% Tlim for example), no relationship between cortical excitability and relative time to fatigue was observed. This would suggest that changes in cortical excitability are more dependent on the absolute number of contractions or the time spent making fatiguing contractions than on time relative to an arbitrary point of task failure (in our case, when MVC fell below 60%), or that activation strategies varied between participants across the task.

We also assessed changes in cortical excitability associated with a protocol that did not elicit central fatigue on the control day to account for changes in cortical excitability that occur in response to the repeated activation of muscle. Studies of fatigue that compare baseline and post fatigue changes in cortical excitability without a control experiment [21,24,44]) may not account for changes in cortical excitability due to activation history alone. In the present investigation, MVC declined by 13% on the control day with no significant change in maximal voluntary activation. Therefore, it is likely that this decline was due to peripheral factors as indicated by the reduction in twitch peak tension. Interestingly, despite the absence of central fatigue we still report changes in cortical circuitry and include a reduction in intracortical and interhemispheric inhibition from baseline to Tlim. This is in line with the notion that reduced interhemispheric inhibition observed following an acute bout of manual training [46] may serve to reduce coupling between hemispheres and promote the ability to manipulate the hands independent of one another. This finding raises an interesting question of whether reduced intracortical inhibition reported previously with fatigue is associated with fatigue or non-fatiguing activation history.

Finally, the ipsilateral motor cortex has been suggested to play an important role in the modulation of motor output to ipsilateral muscles under conditions that present with unilateral motor deficits such as stroke [47]. This has been suggested to occur through ipsilateral corticospinal projections (for review see [48]) or the modulation of contralateral corticospinal output via the interhemispheric inhibitory pathway [49,50]). Previous studies have considered the role of the ipsilateral motor cortex in fatigue and found that ipsilateral motor cortex excitability declines with the contralateral motor cortex following fatiguing unimanual contractions [24,51]. This investigation is the first to assess the role of the ipsilateral motor cortex to compensate for the fatiguing motor cortex via alterations in interhemispheric inhibition. The present study did reveal a progressive decline in MEP amplitude of the non-fatigued ipsilateral motor cortex during the centrally-fatiguing task, however, this reduction in ipsilateral motor cortex excitability was not associated with altered interhemispheric inhibition of the fatiguing contralateral motor cortex. This suggests that the ipsilateral motor cortex did not play a compensatory role to offset reductions in contralateral fatiguing motor cortex excitability via the interhemispheric inhibitory pathway. A more likely explanation is that reduced excitability in the non-fatigued ipsilateral motor cortex may be a consequence of altered interhemispheric transmission from the fatigued motor cortex. It is also possible that bilateral reductions in motor cortex excitability may also be due to reduced drive from upstream premotor structures that affect both hemispheres.

### 4.2. Effect of 5 Hz rTMS to supplementary motor area during recovery from fatigue

rTMS upstream to the primary motor cortex was associated with greater force production immediately after stimulation. While the possibility that this was a placebo response to stimulation cannot be ruled out, it is also possible that offsetting a fatigue-associated decline in premotor activity [18,19] using high frequency rTMS to the supplementary motor area decreased sense of effort and to facilitate maximal voluntary force production. In support of this, we found a strong correlation between intracortical facilitation and sense of force that was not present in the absence of SMA stimulation. Specifically, higher levels of intracortical facilitation were associated with lower sense of effort. Although this is a correlation, we speculate that stimulation of the supplementary motor area may have resulted in a global increase in the excitability of facilitatory circuits within the hand region of the primary motor cortex that in turn may have reduced the amount of upstream drive required to maintain motor output from the primary motor cortex. It is therefore possible that this relationship may have contributed to enhancement of recovery of the MVC.

Although rTMS altered the relationship between intracortical facilitation and sense of effort, it did not elicit the hypothesized increase in MEP amplitude or directly reduce voluntary activation failure. This may have been due to either coil orientation or the time course of the effects of rTMS protocol employed with respect to recovery from fatigue. In contrast to other rTMS protocols used to alter supplementary motor area activity [33,37,38], rTMS was applied to the supplementary motor area with the coil handle oriented posteriorly in this investigation. The rationale for this coil orientation was to stimulate both hemispheres in attempt to offset the bilateral reduction in supplementary motor area activity observed following unilateral fatigue [19]. It is also possible that stimulation of both hemispheres of the supplementary motor area may have resulted in compensatory effects between hemispheres. Finally, a particular challenge with combining a fatigue protocol with rTMS is the time sensitivity of optimal effects elicited by the rTMS protocol employed and time course of recovery of fatigue-related changes in cortical excitability. Optimal effects of the rTMS protocol employed have been reported to occur around 5 minutes after stimulation and last up to 15 minutes [33]. Furthermore, cortical excitability had recovered following completion of the 5 minute stimulation protocol. Although speculative, it is possible that a more robust effect could have been elicited with alignment of optimal rTMS effects and fatigue-induced changes in cortical excitability. However, it appears that stimulation protocol may have increased the rate of recovery of the maximal force by decreasing sense of effort via increased cortical facilitation- an effect and relationship that did not emerge on the day stimulation was not applied to the SMA. It is of interest to note that the same relationship between intracortical facilitation and sense of effort has been observed following head injury, such that participants with lower intracortical facilitation following head injury have a greater sense of effort during fatiguing muscle contractions [52].

In summary, we provide evidence for changes in circuitry upstream from the corticospinal motor neuron that occur during and following the termination of a fatiguing series of muscle contractions. Coinciding with reductions in voluntary activation during fatiguing muscle contraction is a decline in corticospinal excitability and an increase in intracortical facilitation. At task failure it appears that there are a number of compensatory events upstream from the corticospinal motor neuron that may act to offset downstream failure. This can occur either within the primary motor cortex itself through reductions in GABA_A_-mediated intracortical inhibition and increases in cortical facilitation or by increasing the amount of drive from centres upstream to the motor cortex. We applied high frequency rTMS to the supplementary motor area to enhance the rate of recovery of neuromuscular function following fatigue and found that the ability to generate maximal force recovered more quickly with stimulation. The increase in intracortical facilitation following SMA stimulation was correlated with a decreased sense of effort. This observation is consistent with the hypothesis that higher levels of cortical facilitation may reduce the amount of upstream drive required to activate muscle and generate maximal force thus altering sense of effort.

## Supporting Information

S1 Fig Voluntary activation, test MEP, ipsilateral MEP, intracortical facilitation, intracortical, and interhemispheric inhibition datasets to accompany Fig 4. (XLSX)

S2 Fig Maximal torque, maximal voluntary activation, and test MEP amplitude datasets to accompany Fig 5. (XLSX)

S3 Fig Intracortical facilitation and first (a1) and second (a2) sense of force rate constant dataset to accompany Fig 6. (XLSX)

S1 Table Resting (BiStim and Rapid) and Pre-contraction motor threshold datasets to accompany Table 1. (XLSX)

S2 Table Stimulator output and MEP amplitude for test and conditioning pulse datasets to accompany Table 2. (XLSX)

S3 Table Maximal force, EMG, voluntary activation, M wave, muscle twitch characteristics, and cortically-evoked potentials at baseline and post-fatigue (at Tlim, prior to rTMS application) datasets to accompany Table 3. (XLSX)
